# *In vitro* antifungal activity of essential oils extracted from plants against fluconazole-susceptible and -resistant *Candida albicans*

**DOI:** 10.29252/cmm.3.2.1

**Published:** 2017-06

**Authors:** F Katiraee, S Ahmadi Afshar, SF Rahimi Pirmahalleh, H Shokri

**Affiliations:** 1 Department of Pathobiology, Faculty of Veterinary Medicine, University of Tabriz, Tabriz, Iran; 2 Department of Pathobiology, Faculty of Veterinary Medicine, Amol University of Special Modern Technologies, Amol, Iran

**Keywords:** Antifungal resistance, *Candida albicans*, Essential oils

## Abstract

**Background and Purpose:**

*Candida* *albicans* is the most common cause of candidal infections. Various studies have shown drug resistance among *C. albicans* isolates; thus, it is necessary to discover replacement treatments for *Candida* infections. In this study, we aimed to compare the effects of different essential oils against azoles-resistant and azoles-susceptible isolates.

**Materials and Methods:**

Twenty fluconazole-resistant and 20 susceptible *C. albicans* isolates obtained from oral, vaginal, and cutaneous tissues of patients with candidiasis were evaluated. The efficacy and minimum inhibitory concentrations (MICs) of *Zataria multiflora*, *Geranium herbarum, ****Lavendula officinalis****, Cuminum, cyminum,* *Allium heamanthoides,* and *Artemisia sieberi* essential oils against *C. albicans* were determined on the basis of a reference method for broth microdilution susceptibility testing of yeasts as suggested by Clinical and Laboratory Standards Institute (CLSI, M27-S4). After inoculation, incubation, and subculturation, the MICs were determined through comparison with the control.

**Results:**

The obtained MICs for *Zataria multiflora*, *Geranium herbarum*, *Artemisia sieberi*, *Allium heamanthoides*, *Cumminum cyminum*, and *Lavendula officinalis* were 0.1-0.25 µl/ml (mean: 0.155 µl/ml), 0.625-1.66 µl/ml (mean: 0.93 µl/ml) 0.833-2.0 µl/ml (mean: 1.21 µl/ml), 0.1-0.25 µl/ml (mean: 0.155 µl/ml), 2-4 µl/ml (mean: 3.1 µl/ml), and 1.5-3.0 µl/ml (mean: 2.4 µl/ml), respectively. The results showed that *Zataria multiflora* and *Allium heamanthoides* essential oils were more efficient than other essential oils against *Candida* species. There were no significant differences between various *Candida* strains in terms of susceptibility to the essential oils. In addition, there were no significant differences in the MICs of these essential oils against the azoles-resistant and azoles-susceptible isolates.

**Conclusion:**

In this study, the anti-*Candida* effects of six essential oils against both azoles-resistant and azoles-susceptible isolates were similar. Given the documented resistance of different *Candida *species to synthetic and chemical antifungals, these essential oils are effective replacement treatments for cutaneous and mucosal *Candida* infections, especially in resistant or recurrent cases.

## Introduction

*Candida* species are the most prominent agents of fungal infections in humans and animals. These infections are more common in patients with such predisposing factors as cancer, leukemia, diabetes mellitus, long-term antibiotic and corticosteroid therapy, AIDS, pregnancy, burns injuries, and organ transplantation. The spectrum of these infections varies from mucosal colonization to invasive and fatal infections. Cutaneous and mucosal candidiasis is more common than other clinical *Candida* infections. Vaginal candidiasis, thrush, and onychomycosis are the most common types of mucosal and cutaneous candidiasis. In addition, *Candida albicans* is the most prevalent agent of candidal infections among *Candida* species [1-4]. 

Until recently, various antifungal drugs including echinocandiens, azoles (e.g., fluconazole), and polyens (e.g., nystatin) have been used for the treatment of *Candida* infections; however, currently, multiple studies have demonstrated *Candida* species are resistant to a wide range of antifungals, and infections in many cases are attributable to these drug resistant species, especially in immunocompromised patients [2-4]. Replacement treatments are therefore necessary for eliminating drug resistant infections and the undesirable side effects of synthetic and chemical drugs, as well as preventing recurrent infections. 

Humans have used traditional medicines and herbal drugs for thousands of years for the treatment of various diseases. In the nineteenth century, herbal drugs were replaced with chemical compounds. Currently, based on the development of drug resistant strains of various bacteria, viruses, fungi, and organisms, scientists are reconsidering the potential efficacy of traditional medicines and herbal drugs. 

In Iran, traditional medicines have been studied by scientists such as Avicenna and Jabir Ibne Hayyan, and this field remains important to Iranian researchers. Further, multiple studies have exhibited the antimicrobial and antifungal activity of native Iranian herbal drugs such as *Zataria*, *Geranium*, ***Lavendula,*** and *Artemisia* [5]. In the present study, we compared the susceptibility of azoles-resistant and azoles-susceptible isolates to six herbal essential oils and determined whether azoles-resistant isolates show comparable resistance to herbal essential oils. 

## Materials and Methods

***Candida strains***

From different medical laboratories in Tehran, Iran, we acquired 40 *Candida* *albicans *isolates obtained from human infections. Among the 40 *C.* *albicans *isolates*,* 15 were oral, 15 vaginal, and 10 cutaneous clinical isolates. These isolates were determined based on diagnostic methods such as germ tube production, colony color on CHROMagar *Candida*, and carbohydrates assimilation (RapID Yeast Plus System) and were confirmed by a molecular technique in previous studies [6].

***Extraction of essential oils***

Sample plants were collected from different regions of Iran. The plants were ground to a powder in a mill. The prepared powder was kept in tight containers completely protected from light. The essential oil was obtained by hydrodistillation of 300 g of the powder for 4 h using a Clevenger-type apparatus to collect the oil [7].

***Antifungal susceptibility testing***

Susceptibility to fluconazole was determined on the basis of a reference method for broth microdilution susceptibility testing of yeasts, which was suggested by the Clinical and Laboratory Standards Institute (CLSI -M27-S4) [8]. 

The evaluation of susceptibility to essential oils was performed according to broth microdilution such as fluconazole. A stock solution of essential oils was prepared by adding 0.1 ml of each essential oil to 1.25 ml dimethyl sulfoxide (DMSO), its volume was increased to 10 ml using RPMI 1640 (Stock 1% V/V). Thereafter, serial dilutions were prepared from the stock solution in microplate wells. 

After a pilot study, for evaluation of anti-*Candida* activity, *Zataria multiflora *and* Alium* were used at 0.08-2 µl/ml concentrations, while *Geranium*, *Artemisia, ****Lavendula,*** and* Cumminum* were utilized at 0.2-5 µl/ml concentrations. The diluted microplates, after inoculation with organisms, were incubated at 35°C for 48 h. After incubation, the plates were evaluated with regards to fungal growth, that is, each well was subcultured on SDA, and after 48 h, it was examined for fungal growth and colony counting was performed. To determine MIC, colony count of each well was compared to that of blank and positive control wells.

***Gas chromatography-mass spectrometry (GC-MS) analysis***

The essential oils were analyzed by GC-MS. The chromatograph instrument (Agilent 6890, UK) was equipped with an HP-5ms capillary column (30 × 0.2 mm ID × 0.2 μm film thickness) and the results were taken under the following conditions: initial temperature at 50°C, temperature ramp at 5°C/min, 240°C/min to 300°C (holding for 3 min), and injector temperature at 290°C. The carrier gas was helium and the split ratio was 0.8 ml/min. For confirmation of the analysis results, the essential oils were also analyzed by GC-MS (Agilent 6890 gas chromatograph equipped with an Agilent 5973 mass-selective detector; Agilent, UK) using the same capillary column and analytical conditions as above. Mass spectrometry was performed in electron-ionization mode with ionization energy of 70 eV. 

## Results

The results of GC-MS analysis of the essential oils are presented in detail in Table 1 The obtained MICs for *Zataria multiflora*, *Geranium herbarum*, *Artemisia sieberi*, *Allium heamanthoides, Cumminum cyminum,* and ***Lavendula officinalis*** were 0.1-0.25 µl/ml (mean: 0.155 µl/ml), 0.625-1.66 µl/ml (mean: 0.93 µl/ml) 0.833-2.0 µl/ml (mean: 1.21 µl/ml), 0.1-0.25 µl/ml (mean: 0.155 µl/ml), 2-4 µl/ml (mean: 3.1 µl/ml), and 1.5-3.0 µl/ml (mean: 2.4 µl/ml), respectively. The results showed that *Zataria multiflora *and* Allium* were more efficient than other essential oils against*C. albicans*. There were no significant differences between susceptibility of different *C.* *albicans* to the six essential oils. In addition, there were no significant differences in the MICs of these essential oils against the azoles-resistant and azoles- susceptible isolates. Based on the source of isolation of *Candida* infection, we could not find any significant differences between susceptibility of different *C. albicans* to the six essential oils. (table 2 and 3).

**Table 1:** The main components of six essential oils identified by gas chromatography-mass spectrometry analysis

**No**	***Zataria multiflora***	***Geranium herbarum***	***Artemisia sieberi***	*Lavendula officinalis*	***Cuminum, cyminum***	***Allium heamanthoides***
1	Thymol	Citronellol	Santolina alcohol	g-terpinene	1,8-cineole	Diallyl disulfide
2	Carvacrol	Geraniol	Oxygeneted monoterpenes	Cuminaldehyde	Cuminaldehyde	Trisulfide, methyl 2-propenyl
3	γ-Terpinene	- Bourbonene	Camphor	2-norpinene-2-carboxaldehyde	Cyclopentapyran	Trisulfide, di-2-propenyl
4	p-Cymene	Menthone	Camphene	b-pinene	O-cymene	Disulfide, methyl 1-propenyl
5	Methyl ether Carvacrol	Geranyl formate	a-thujone	Benzene methanol	2b-pinene	Dimethyl trisulfide
6	α-Pinene	Citronellyl propionate	b-thujone	O-cymene O-cymene	α-Pinene-7-ol	2-Chlorbenzothiazole
7	trans-Caryophyllene	Linalool	cymene	Pulegone	Moslene	Tetrasulfide, di-2-propenyl
8	Linalool	Citronellyl acetate	α-Terpineole	Sabinene	d-3-carene	3-Vinyl-1,2-dithiacyclohex-4-ene
9	α-Terpinene	0-octen-1-0	Sabinol (cis)	b-myrcene	g-terpinene	3-Vinyl-1,2-dithiacyclohex-5-ene
10	Myrcene	Caryophyllene oxide	1,8 Cineole	a-thujene	Myrcene	Pentanoic acid

**Table 2:** The obtained minimum inhibitory concentrations for Zataria multiflora, Geranium herbarum, Artemisia sieberi, Allium, Cumminum cyminum, and Lavendula officinalis based on source of isolation

**Sign**	**Max**	**Min**	**Means**	**Source of isolation(n)**	**Essential oils**
	0.25	0.1	0.142	Oral(15)	*Zataria multiflora*
N	0.25	0.125	0.165	Vaginal(15)	
	0.25	0.125	0.17	Cutaneous(10)	
	1.66	0.625	1.0	Oral(15)	*Geranium herbarum*
N	1.25	0.625	0.904	Vaginal(15)	
	1.25	0.625	0.883	Cutaneous(10)	
	1.66	1.0	1.291	Oral(15)	*Artemisia sieberi*
N	1.66	0.833	1.173	Vaginal(15)	
	2.0	0.833	1.174	Cutaneous(10)	
	0.25	0.1	0.14	Oral(15)	*Allium heamanthoides*
N	0.25	0.1	0.165	Vaginal(15)	
	0.25	0.125	0.17	Cutaneous(10)	
	4.0	2.0	3.05	Oral(15)	*Cumminum cyminum *
N	4.0	2.0	3.2	Vaginal(15)	
	4.0	2.0	3.15	Cutaneous(10)	
N	3.0	1.5	2.55	Oral(15)	***Lavendula officinalis***
	3.0	1.5	2.6	Vaginal(15)	
	3.5	1.5	2.35	Cutaneous(10)	

N: non-significant

**Table 3:** The obtained minimum inhibitory concentrations for Zataria multiflora, Geranium herbarum, Artemisia sieberi, Allium, Cumminum cyminum, and Lavendula officinalis based on susceptibility of strains to fluconazole

**Sign**	**Max**	**Min**	**MIC90**	**MIC50**	**Means**	**Susceptibility to fluconazole(n)**	**Essential oils**
N	0.25	0.1	0.2	0.15	0.161	Resistance(20)	*Zataria multiflora*
	0.25	0.1	0.175	0.15	0.156	Susceptible(20)	
N	1.25	0.625	1.0	0.833	0.874	Resistance(20)	*Geranium herbarum*
	1.66	0.625	1.25	1.0	0.985	Susceptible(20)	
N	2.0	0.833	1.5	1.25	1.21	Resistance(20)	*Artemisia sieberi*
	1.66	0.833	1.5	1.25	1.215	Susceptible(20)	
N	0.25	0.1	0.175	0.15	0.165	Resistance(20)	*Allium* *heamanthoides*
	0.25	0.1	0.15	0.15	0.152	Susceptible(20)	
N	4.0	2.0	3.5	3.0	3.033	Resistance(20)	*Cumminum cyminum *
	4.0	2.0	4.0	3.0	3.233	Susceptible(20)	
N	3.0	1.5	3.0	2.5	2.2	Resistance(20)	***Lavendula officinalis***
	3.5	1.5	3.0	3.0	2.6	Susceptible(20)	

N: non-significant

## Discussion

Azole drugs have fungistatic activity via interference with the synthesis of fungal ergostrol. These broad-spectrum drugs are frequently used for the clinical treatment of microbial and fungal infections. The mechanism of action of these drugs involves interference with certain human functional pathways; therefore, they have important side effects on the human body [1, 9]. The development of drug resistance among *Candida *species in clinical infections isa problem physicians commonly encounter. This problem is especially evident in patients with candidal infections who previously used azoles such as fluconazole and ketoconazole. In immunocompromised patients (e.g., AIDS), candidal infections are common and the use of fluconazole is the current treatment, but recurrent infections are highly common in this population. Moreover, azole antifungals are used for the treatment of vaginal and cutaneous candidiasis and most isolates are not susceptible to this drug. Thus, finding replacement treatments are important for the patients suffering from refractory or recurrent candidiasis [2, 4]. 

Several pharmacological properties, such as antimicrobial, antifungal, anti-seizure, anti-nociceptive, anti-*Candida*, anti-septic, anti-aphtous, analgesic, carminative, and anti-inflammatory effects, have been reported for *Zataria multiflora*. It has been reported that Z. multiflora essential oil can stimulate innate immunity and has antibacterial, antifungal, and antioxidant activities [5, 10]. Antimicrobial activity of *Zataria multiflora* is related to some compounds such as thymol, rosemaric acid, and carvacrol. Several studies have commented on the antifungal effects and properties of *Zataria* against fungal species [11-15]. 

Results of this study regarding the usage of *Zataria multiflora* are useful because the quantitative determination of MICs of its essential oils is necessary for its application in pharmaceutical products. In addition, the results of this study indicated that *Zataria* *multiflora* essential oil can be an appropriate alternative for chemical drugs as no significant difference in susceptibility to *Zataria* *multiflora* between drug-resistant and susceptible isolates was observed.

The essential oil of *Geranium* plant is used in wound healing and as a purgative agent and analgesic to relieve bladder and chest pains. It is applied in the treatment of diabetes, pulmonary hemorrhages, gout, sore throat, hypertension, as an ointment in the treatment of burns injuries. Antifungal effects of *Geranium,* especially on *Fusarium*, *Aspergillus*, and *Saprolegnia,* have been explained, and different studies demonstrated diverse antibacterial effects of its essential oil [16, 17]. 

The results of this study indicated the anti-candidiasis effects of *Geranium* essential oil; according to the comparisons between MICs against azole-resistant and azole-susceptible isolates, *Geranium* *herbarum *can be used for the treatment of recurrent and treatment-resistant *Candida* infections.

*Artemisia* is a one- to multi-year-old plant fromthe *Anthemideae *family (sunflower family). It has nutritional value for livestock and it is known as one of the popular forage, especially in desert areas. *Artemisia* is applied for the treatment of fungal infections. The ointment obtained from this plant is highly effectivein treating superficial fungal infections such as dermatophitosis and *Malassezia* infections [18]. 

The results of the current study exhibited the anti-fungal effect of *Artemisia* essential oil. Furthermore, due to the lack of difference in the MIC of the studied essential oils against azole-resistant and azole-susceptible *Candida* species, it can be used in treating recurrent and treatment-resistant *Candida* infections*.*

*Allium *is the largest and most important representative genus of Alliaceae family. Several biological activities have been reported for *Allium *species such as antibacterial, antiviral, anti-parasitic, antifungal, anti-protozoa, anti-diabetic, antioxidant, and anti-carcinogenic effects, as well as effects on the circulatory and cardiovascular system and treatment of common cold [19]. Many of the biological effects of *Allium *species are related to thiosulfate and volatile sulfur compounds typical of these plants, which are also responsible for their pungent aroma and taste properties [19, 20]. In-vitro studies have confirmed the ability of some *Allium heamanthoides* plants to reduce the parameters accounted for as risk factors for cardiovascular diseases such as raised total serum cholesterol, elevated low-density lipoprotein, increased platelet aggregation, hypertension, and smoking [21].

Our results showed the anti-candidiasis effects of *Allium* *heamanthoides* essential oil. Based on the results of the comparisons between MICs against azole-resistant and azole-susceptible isolates, *Allium heamanthoides* can be used for the treatment of recurrent and resistant infections.

Lavender is an evergreen shrub from the Lamiaceae family. Lavender oil is consisted of more than 100 compounds, but linalool and linalylacetate are the most important ones [22, 23]**. **Pharma-ceutically, this plant has antispasmodic, sedative, and perspiration stimulant effects, and it is used in treating common cold, flu, and digestive problems such as colic [13, 24]. Further, flowers of these plants have carminative and tonic properties. The essential oil extracted from this plant’s flowers is used topically in rheumatic pain. Lavender oil has been traditionally used as an antiseptic agent for burns injuriesand parasite bites. It also has antifungal effects against *Aspergillus nidulans* and *Trichophyton mentagrophytes* [25]. Our results demonstrated the anti-candidiasis effects of lavender essential oil; according to the results of the comparisons between MICs against azole-resistant and azole-susceptible isolates, lavender can be used for the treatment of recurrent and resistant infections. 

*Cuminum cyminum *L. (Cumin) originates from the Mediterranean region and is now cultivated in many parts of Asia such as Iran [26-28]. Traditionally, cumin has been used to treat jaundice, dyspepsia, and diarrhea. It also has carminative, stimulant, diuretic, and astringent effects [26, 29]. It has long been utilized in food preservation because of its antimicrobial effects on microorganisms [30-33]*.*

It is also effective in the growth of bacteria commonly employed in the food industry such as *Lactobacillus curvatus* and *Staphylococcus xylosus*. Moreover, it has inhibitory effects on the growth of some fungi such as *Aspergillus niger* and *C. albicans *[34, 35]. The essential oil extracted from cumin also has fungicidal effect on *Aspergiluus flavus *[36]. Use of supplements containing *Cuminum cyminum* in diabetic rats reduced fatty change and inflammatory cell infiltrates. It was also more effective than glibenclamide in treating diabetes mellitus. Cumin can modulate carcinogen metabolism, therefore, it is believed to have strong chemoprotective potential. As for immunological effects, *Cuminum cyminum *stimulated the T cells and Th1 cytokines expression in normal animals [37]. 

According to significant anti-candidal effects of the reviewed essential oils, lack of resistance against these compounds, and the increase in outbreaks of drug resistance among candidal organisms, it seems that therapeutic effects of these compounds can be useful. The reviewed plants grow widely in fields of Iran, which makes them easier to use. Another point is determination of MIC for these essential oils since the direct application of these compounds on the skin and mucous is impossible due to the potential harms, they need to be used as mixtures with other effective compounds. Given the obtained MICs, the essential oil of *Zataria* had the most significant effect and a small amount of this essential oil in drug compounds seems to be useful, although this finding needs more deliberation. However, future studies should investigate simultaneous usage of these natural compounds and chemical drugs and overcoming drug resistance through using herbal medicines. Further, they should study whether long-term application of these essential oils can result in resistance or side effects.

## Conclusion

*Z. multiflora* and *A. heamanthoides* essential oils showed high antifungal activity when compared with other essential oils. In this study, no differences in susceptibility between *C. albicans* strains were noted, indicating that the resistant and susceptible *C. albicans* have similar susceptibilities to these essential oils. With reference to the considerable effects of the examined herbal essential oils on drug resistant and susceptible *Candida* isolates, the use of these natural compounds is recommended over chemical drugs. We suggest using these compounds to counter both azoles-resistant strains and recurrent infections.
